# An Incidental Finding of Morgagni Hernia in an Elderly Female and Its Successful Management: A Rare Case Report and Review of Literature

**DOI:** 10.7759/cureus.42676

**Published:** 2023-07-30

**Authors:** Aditya Sharma, Rahul Khanna, Panchanana Panigrahy, Ram Niwas Meena, Shashi Prakash Mishra, Seema Khanna

**Affiliations:** 1 Department of General Surgery, Institute of Medical Sciences, Banaras Hindu University, Varanasi, IND

**Keywords:** rare case report, roof top incision, chilaiditi sign, symptomatic morgagni hernia, adult morgagni hernia

## Abstract

Morgagni hernia is one of the congenital diaphragmatic hernias, but it can also occur in adults. It usually manifests in childhood, but in rare cases, it may also present in adults. It is linked to a congenital defect during the embryological development of the diaphragm. Uncommon diaphragmatic hernias, also called the foramen of Morgagni hernias, often affect the right side and are found in the anterior mediastinum.

Typically asymptomatic in adult patients, the foramen of Morgagni hernia is linked to obesity, trauma, or other causes of elevated intraabdominal pressure. Diagnostic aids include plain pulmonary roentgenograms, contrast-enhanced radiographic investigations of the gastrointestinal tract, computerised tomography, and magnetic resonance imaging tests.

We report a rare case of an 85-year-old female with a Morgagni hernia that was incidentally detected on a chest X-ray and was managed successfully.

## Introduction

Diaphragmatic hernias of Morgagni are structural abnormalities in the anterior diaphragm that permit herniation of abdominal viscera into the thorax [1]. They are the most uncommon congenital diaphragmatic hernias and account for only 2-3% of cases [2]. Only 12 cases of symptomatic Morgagni hernias in adults have been documented, the majority of them manifesting as abdominal agony brought on by strangulation of the viscera [3]. The omentum, small bowel, or stomach are the herniated viscera in all such symptomatic cases [4].

## Case presentation

An 85-year-old woman presented with the chief complaint of pain in the abdomen for 20 days associated with a single episode of vomiting along with non-passage of flatulence and stools for the past seven days. It was associated with a single episode of vomiting that was non-projectile, non-bilious, and contained food particles. There was also a history of non-passage of flatus and stools, as stated. There was no history of fever, jaundice, hematemesis, or melena. She has had a known case of hypertension for the past seven years, for which she is taking anti-hypertensive drugs.

On abdominal examination, the abdomen was flabby, and the umbilicus was centrally placed and everted. On palpation, the abdomen was soft and non-tender, and on digital rectal examination, there were no anal tags, normal anal tone, smooth rectal mucosa, any obvious growth, or any ballooning or collapsed rectal wall. All haematological and biochemical parameters were within normal limits. The patient’s chest X-ray showed an elevated right dome of the diaphragm with a bowel containing gas shadow (Chilaiditi sign).

On ultrasonography (USG), the upper quadrant of the abdomen showed large bowel loops between the diaphragm and the superior surface of the liver, with sluggish peristalsis. Contrast-enhanced computed tomography (CECT) suggested a large antero-medial right diaphragmatic hernia with a defect of 8 cm × 3 cm; the size of the hernial sac was approximated as 14 cm × 13 cm × 8 cm; and the contents of the sac included hepatic flexure of the colon, omental fat, and the pyloric region of the stomach, features suggestive of an obstructed right diaphragmatic hernia.

**Figure 1:**
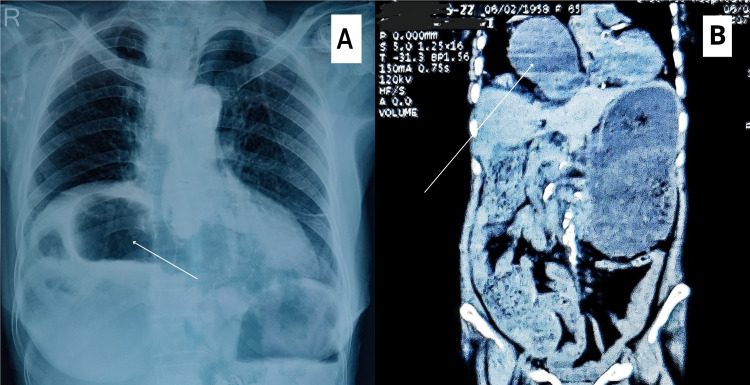
(A) A X-ray thorax film showing the Chilaiditi sign, i.e., the interposition of the bowel, usually colon, between the inferior surface of the right hemidiaphragm and the superior surface of the liver; (B) a CECT thorax and whole abdomen film showing a large antero-medial right diaphragmatic hernia with bowel herniating through the defect.

The X-ray thorax film showing the elevated right dome of the diaphragm with bowel-contained shadow and the CECT film are shown in Figure 1.

Several surgical principles must be followed in order to successfully treat Morgagni's hernia. To fully reveal the hernia defect, the falciform ligament should be completely removed since it may conceal the full extent of the fascial defect. By removing this ligament, an additional space is created. Small defects can usually be repaired without tension.

Depending on the surgeon's preference, the surgeon may choose to do a primary repair of the hernia in addition to repairing the diaphragm edge to the anterior abdominal wall using permanent sutures. Abdominal contents herniate via an anterior retrosternal defect to form the foramen of Morgagni's hernia. Contrary to Bochdalek's hernias, anterior defects in the retrosternal region are frequently accompanied by a big hernia sac, allowing for the herniation of a sizable amount of abdominal content before the patient is prepared to undergo surgery. In addition to hollow and solid viscera that have been incarcerated for a long time, the intrathoracic hernia sac often includes the omentum and falciform ligament.

In the present case, the patient was scheduled for an open diaphragmatic hernia repair. A roof-top incision was made. Intraoperatively, parts of the transverse colon, pylorus, and omentum were seen herniating into the thoracic cavity through the defect. The contents were reduced, and a defect of 6 cm × 3 cm was seen in the right dome of the diaphragm. The defect was repaired by doing the primary repair.

**Figure 2:**
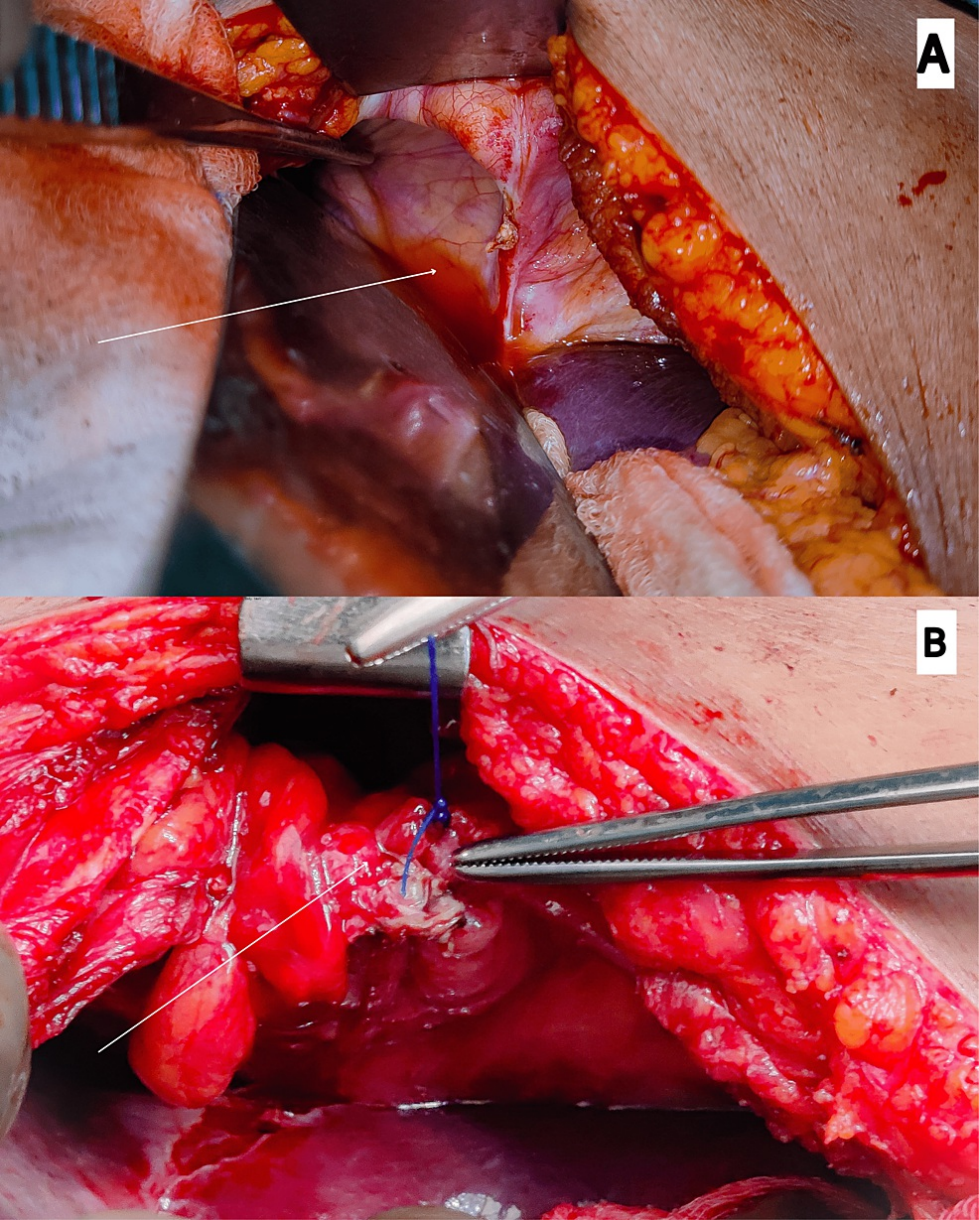
(A) An intraoperative picture showing a defect of 6 cm × 3 cm in the right dome of the diaphragm post reduction of hernia contents. (B) Another intraoperative picture showing the primary closure of the defect done successfully.

The intraoperative pictures showing the defect in the right dome of the diaphragm post-reduction of the hernia contents and primary closure of the defect have been shown in Figure 2.

The patient was allowed oral intake on postoperative day 1, which she tolerated well. The patient did well during the hospitalization period and was discharged on postoperative day 3 under satisfactory conditions.

## Discussion

Patients with a diaphragmatic hernia typically present with respiratory symptoms when they are younger. Adult patients rarely present with chest symptoms; instead, most of them will complain of abdominal pain attributed to the strangulation of the viscera [5]. The omentum, small bowel, or stomach are the herniated viscera in symptomatic adult patients. It is unclear how diaphragmatic hernias develop [6]. Patients who had previously normal radiographs indicate that these hernias might have developed as a result of a diaphragm abnormality that was present at birth [7].

Because patients may be asymptomatic or exhibit gastrointestinal and respiratory symptoms, the majority of Morgagni hernias are discovered after they have progressed [8]. While CECT is the most sensitive investigation because it provides excellent anatomical detail on the contents of the hernia and has been proven to be effective in assessing diaphragmatic hernias, ultrasonography has also been demonstrated to be beneficial in such cases.

A contentious technical part of Morgagni's hernia surgery still involves the removal of the hernia sac. Early research revealed that by removing the entire sac, the chance of recurrence was reduced by removing the prior lead point. There have also been reports of significant symptomatic effusions necessitating surgical intervention when the hernia sac is left in place [9].

Studies have, however, also documented some negative effects of sac excision, such as pneumothorax and iatrogenic lung or mediastinum damage. However, both the intrathoracic organs and the epigastric vessels should lie outside the plane of the actual hernia sac. Furthermore, injury to the superior epigastric veins has also been mentioned as a potential hazard [10].

Future research should compare laparoscopic and open techniques for treating Morgagni's hernias. This article and the literature do not provide enough support for this claim. Laparoscopic suturing may be suitable for some surgeons ergonomically; however, it is unclear if this benefit offsets potential increases in cost and procedure time.

## Conclusions

Primary surgical repair, which can be performed either trans-thoracically or trans-abdominally, is the treatment of choice for Morgagni hernias. It is recommended that surgical repair be advocated in cases where there is no symptomatic presentation. Even in elderly individuals, the surgical repair of Morgagni's hernia is regarded as the most effective treatment that leads to relief from symptoms and an improvement in quality of life.
